# Novel and Effective Copper–Aluminum Propane Dehydrogenation Catalysts

**DOI:** 10.1002/chem.201102580

**Published:** 2011-09-21

**Authors:** Jana Schäferhans, Santiago Gómez-Quero, Daria V Andreeva, Gadi Rothenberg

**Affiliations:** [a] Van ‘t Hoff Institute for Molecular Sciences, University of AmsterdamScience Park 904, 1098 XH Amsterdam (The Netherlands), Fax: (+31) 20-525-5604; [b] Physical Chemistry II, University of BayreuthUniversitätsstrasse 30, 95444 Bayreuth (Germany)

**Keywords:** heterogeneous catalysis, nanostructures, olefins, propylene, sonochemistry

The importance of solid catalysts for converting petro- and bulk chemicals is reflected in the sheer magnitude of their market size: catalysts’ sales topped nine billion dollars in 2009.^[1]^ This large value mirrors also the increasing academic interest in heterogeneous catalysis research.^[2]^ As far as bulk chemicals (such as ethene, propene, and their derivatives) are concerned, there is a strong demand for clean and inexpensive catalysts and synthesis processes.^[3]^ There are two types of commonly used dehydrogenation catalysts: supported Cr oxides^[4]^ and Pt-based^[5]^ systems. The problem is that these catalysts are typically either rare and costly, or hazardous. Moreover, they perform well only at high temperatures^[6]^ (typically at 500–600 °C) due to thermodynamic limitations.^[7]^ Optimization studies have led to the inclusion of several promoters of which tin, especially in the combination with platinum as Pt–Sn/Al_2_O_3_ is one of the most popular.

We report here the discovery of a new alternative catalyst for propane dehydrogenation which does not contain noble or hazardous metals. It is an oxidized porous Cu–Al alloy with a structure that is similar to Raney-type metals.^[8]^ The Raney process, patented by Murray Raney in 1925 and commercialized by W.R. Grace & Co.,^[9]^ is one of the most successful routes for making porous metals. The problem is that this process requires extreme conditions that often restrict the final outcome at the nanometric scale. Here we opted for a different approach, applying a modified version of the ultrasound pore formation method that we have recently reported: high-power ultrasound.^[10]^

In a typical synthesis, Cu and Al beads are melted by using an electric arc. The resulting cake is then pulverized and sonicated in water. This gives a highly porous material containing pores predominantly at the micro-scale (see Figure 1 a and 1 b for a representative example; catalyst **D**).^[11]^ Figure 1 c and 1 d shows transmission and scanning electron micrographs of such a catalyst. We hypothesize that the sonication creates pores in the Al component followed by surface oxidation (compare the XRD profiles (a) and (b) in Figure 2), whereas Cu supplies the active centers for the catalysis (vide infra). The thickness of the ultra thin oxide layer was estimated by using 3D field ion microscopy as less than 2.0 nm.^[10a]^

**Figure 1:**
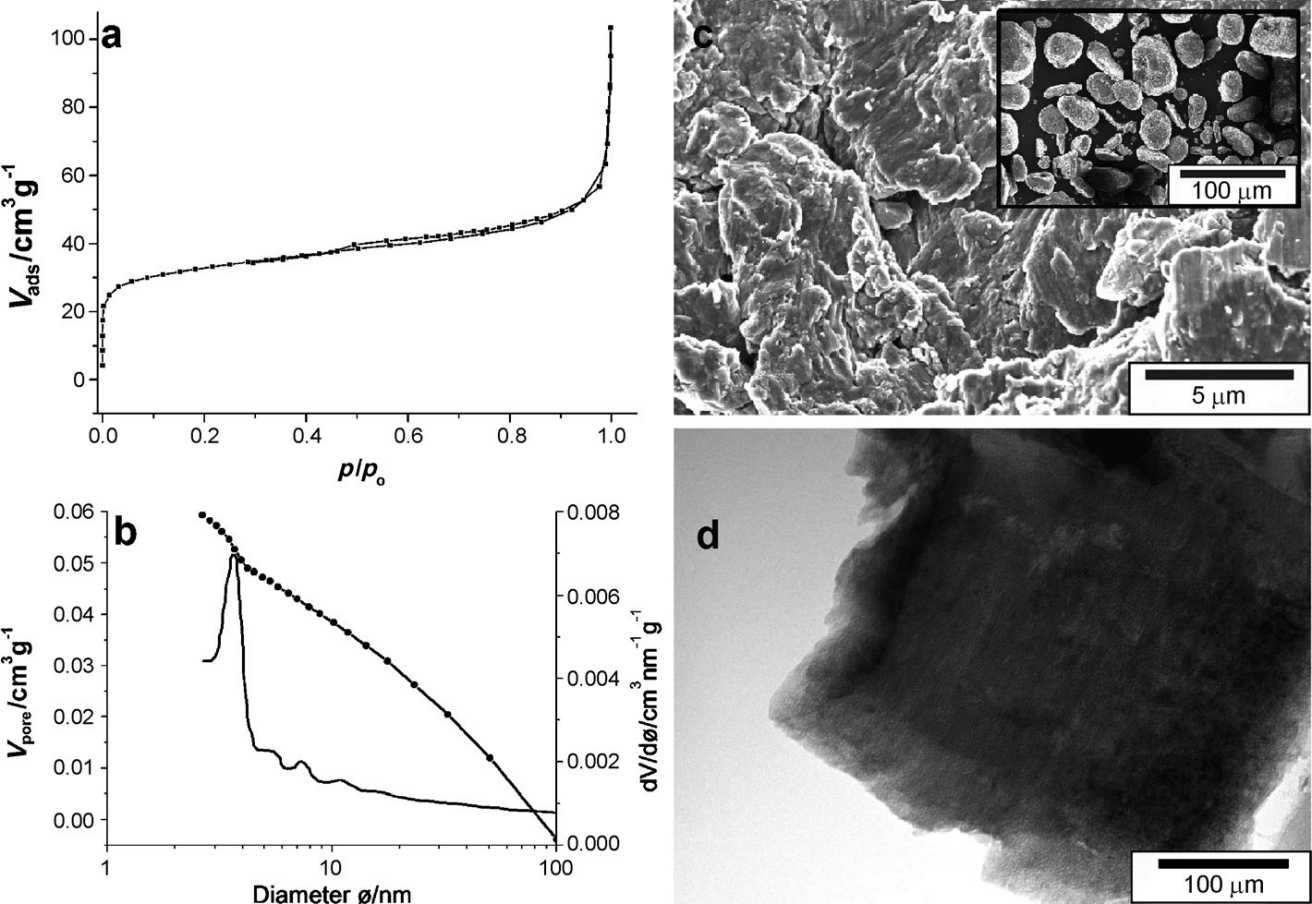
Porous Al–Cu alloy D N_2_ adsorption/desorption isotherms (a), pore size distributions (b), scanning electron micrographs (c) and transmission electron micrograph (d).

**Figure 2:**
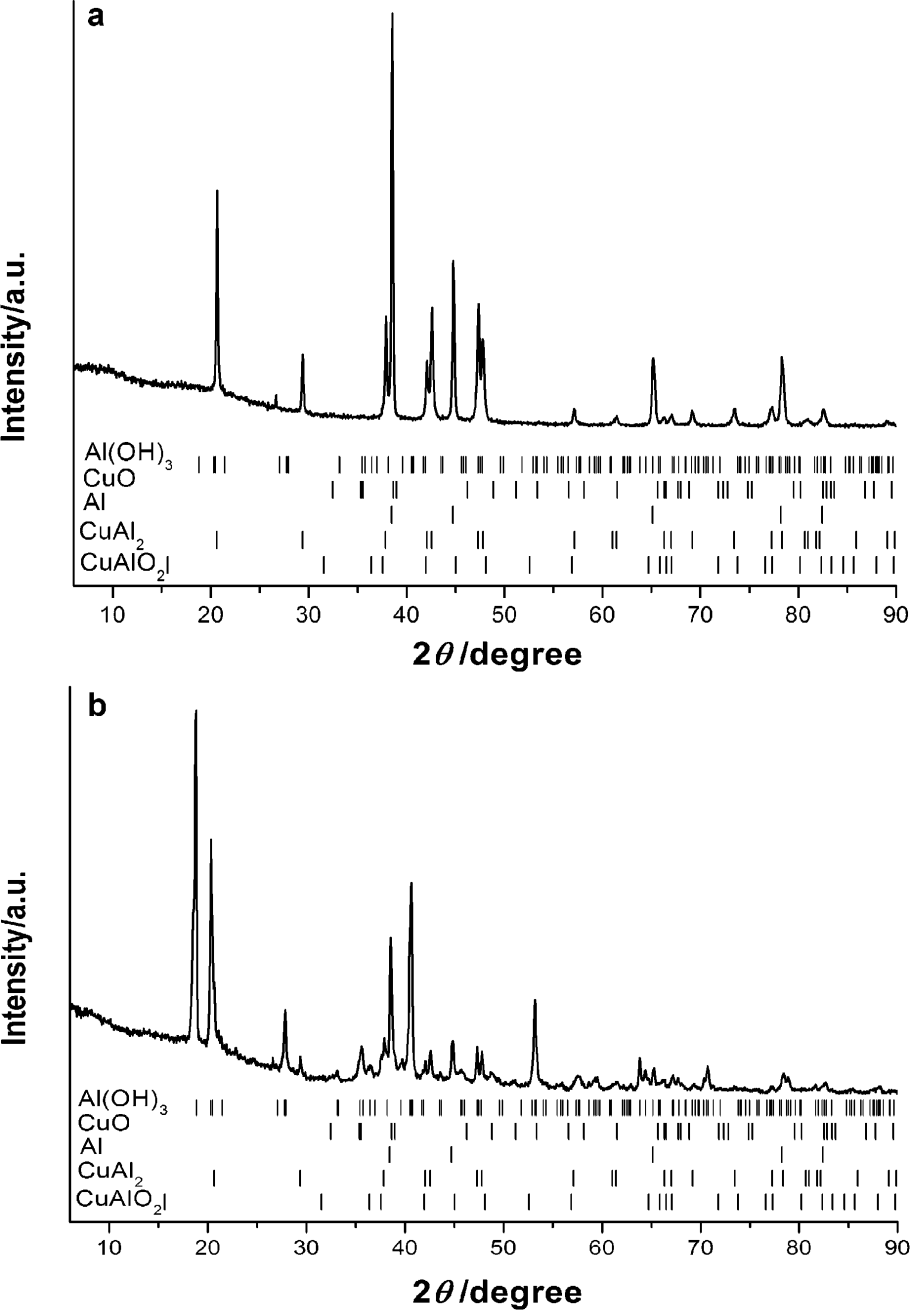
X-ray diffraction patterns of the porous Al-Cu catalyst D before (a) and after (b) ultrasound treatment (the JCPDS-ICDD standards are also included for ease of comparison).

We prepared a series of catalysts with different Cu content (Table 1, entries 1–4), and found that using 25 wt % Cu (catalyst **D**) gave the most promising results. This catalyst was then activated under different conditions in an effort to optimize the preparation recipe (see entries 4–6). We see a reduction of conversion in the first minutes, probably reflecting some initial sintering and coke deposition (see Figure 3).^[7]^, ^[12]^ After this short deactivation period, the catalyst maintains its steady-state activity (all values hereafter refer to the steady-state period). Our catalyst gave reasonable propane consumption rates already at 550 °C (see Table 1). Note that all the reactions gave very good reproducibility (±7 % for different samples from the same catalyst batch). However, if we look at the theoretical phase diagram of Al–Cu, we see that it shows an eutectic point at 548 °C.^[13]^ True, our catalyst is not a pure Al–Cu alloy (since at least its surface is passivated with an oxide layer; see Figure 2). Nevertheless, we hypothesized that a partial melting occurs during the pre-treatment at 600 °C (and possibly even during the reaction at 550 °C). Even if only part of the catalyst were melting, it would be perforce the active part. This is because the first sites that would melt would be the high-energy kinks and breaks where catalysis usually happens.^[14]^ Indeed, when we compared samples **A** and **B** that had less Cu but a larger particle size (typically>150 μm), we saw that these were more active than those with more copper but smaller sizes. To check this hypothesis, we prepared another batch of the same catalyst **D**, but this time activated at 400 °C (all other conditions identical). We then ran the dehydrogenation again, this time 200 degrees lower (i.e. at 350 °C). Excitingly, as Figure 3 shows, this catalyst gave greater conversions, reaching a stable 4 % on stream. This is equivalent to a constant rate of 0.83 mol h^−1^ g^−1^. This result is all the more remarkable considering the temperature difference: a 200 °C offset would be expected to slow down the reaction by approximately an order of magnitude (all other known catalysts are inactive under these conditions). For comparison purposes, we tested a standard Pt–Sn/Al_2_O_3_ catalyst under similar conditions. This catalyst has been shown in the available literature as the best in terms of activity/selectivity/stability for propane dehydrogenation.^[15]^ Under the same reaction conditions, Pt–Sn/Al_2_O_3_ was practically inactive at 350 °C and gave less than 1 % conversion (<0.2 mol h^−1^ g^−1^) at 550 °C. Searching the literature, we did not find any reports on propane dehydrogenation over Cu/Al_2_O_3_. But, we note the increase in rate quoted by Sokolova et al.^[16]^ when adding Cu to Pt/Al_2_O_3_.

**Table 1:** Composition, surface area and initial dehydrogenation rate for Al–Cu catalysts A–D.

Entry	Catalyst	Cu [wt %]	BET [m^2^ g^−1^]	Initial rate [mol h^−1^ g^−1^]	Activation atmosphere
1[a]	**A**	5	48	1.35	O_2_
2^[a]^	**B**	10	45	1.73	O_2_
3	**C**	15	42	0.20	O_2_
4	**D**	25	34	0.79	O_2_
5	**D**	25	34	0.54	H_2_
6	**D**	25	34	0.01	Ar
7^[b]^	**D**	25	34	0.27	O_2_
8^[c]^	**D**	25	34	3.39	O_2_

[a] Particle size >150 μm.

[b] Reaction run without steam.

[c] Catalyst activation at 400 °C, reaction at 350 °C.

**Figure 3:**
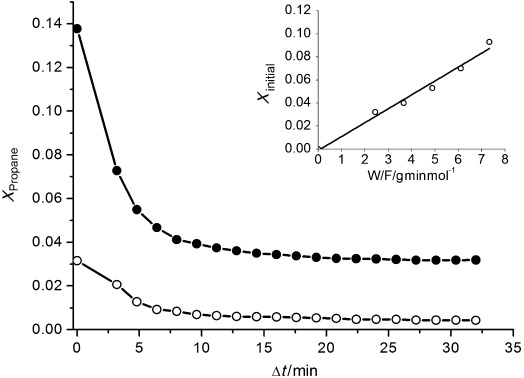
Temporal propane conversion for catalyst **D** at 350 °C (•) and 550 °C (○). Inset: relationship between the catalyst space time (i.e. amount of catalyst per propane molar flow pass) and initial propane conversion. Note: the benchmark Pt–Sn/Al_2_O_3_ catalyst (not shown) gives <1 % conversion at 550 °C.

In conclusion, we show here that high-power ultrasound is a green chemistry tool for the synthesis of porous copper–aluminum frameworks stabilized by metal oxide. Furthermore, this material is inexpensive (production expenses are approximately 3 € per liter) and the method can be easily scaled-up by using different sonotrodes (or a series of them), as these may vary widely in size and shape. These new porous materials (or “metal sponges”) have an alloy bulk and an oxidized surface, and can catalyze propane dehydrogenation at low temperatures. Thanks to their high activity and because they contain no noble metals, they open exciting opportunities in low-temperature dehydrogenation catalysis for making bulk chemicals.

## Experimental Section

A detailed description of the materials and instrumentation used in this study, as well as the procedure for preparing the reference Pt–Sn/Al_2_O_3_ catalyst, are given in the Supporting Information.

**Procedure for preparing the Al–Cu alloy:** Commercial Al and Cu beads were alloyed by an arc melting device (Bühler) with a melt stream of 300 A. After reaching a vacuum of 10–5 mbar, 500 mbar Ar were transferred to the reactor. For homogenization, the melt of Cu and Al was turned around three times. Five different alloy samples (30 g each) were prepared. The Cu content in these samples was 25 wt % (118 mmol), 20 wt % (94 mmol), 15 wt % (71 mmol), 10 wt % (47 mmol), and 5 wt % (24 mmol), respectively. The resulting solid was cut in pieces and then grounded by using a rotary mill (PULVERISETTE 14, Fritsch GmbH) with a sieve ring of 1.5 mm. After milling, the powder was sieved with a mesh of 14.

**Procedure for catalyst preparation**: Five grams of the Al–Cu alloy powder were dispersed in ultrapure water (50 mL) and sonicated for 60 min with an ultrasound tip (Hielscher VIP1000 hd instrument; operated at 20 kHz with a maximum output power of 1000 W and a head area of 3.8 cm^2^, equipped with a booster B2–1.2). The maximum intensity was calculated to be 57 W cm^−2^ at a mechanical amplitude of 81 μm. During the treatment the sample was cooled in an ice bath. After the treatment, the sample was dried at 120 °C for 24 h.

**General procedure for propane dehydrogenation**: The catalyst was activated in situ before reaction in a flow of 77 mL min^−1^ Ar and 3 mL min^−1^ O_2_ at 600 °C. The reactions were carried out at 1 atm and 550 °C in a continuous-flow fixed-bed vertical quartz reactor (4 mm i.d.), which was controlled with a fully automated system built in house.^[14c]^.The partial pressures of C_3_H_8_ and Ar were fixed at 0.5 atm where 2 g h^−1^ of steam were supplied by means of Bronkhorst mass flow controllers (total flow=80 cm^3^ min^−1^); the ratio of catalyst mass (W) to initial C_3_H_8_ molar flow rate (F) spanned the range 0–9 g min mol^−1^. A quartz wool layer (≤ 3 mm) above the catalyst served as a preheating zone and isothermal conditions were kept by diluting the catalyst bed with ground glass; the reaction temperature was continuously monitored by a thermocouple at the catalyst bed. The reactor effluent was analyzed on-line by using an Interscience Compact GC equipped with two TCD detectors separating a) Ar, H_2_O, CO_2_, C_2_ and C_3_ hydrocarbons on a Porabond Q column (helium as carrier gas); and b) H_2_, CO, CH_4_ and O_2_ on a 5 Å molsieve column (argon as carrier gas). The measurement analytical repeatability was better than ±0.5 %. The fractional conversion of C_3_H_8_ is defined in this study as X_A_=((F−F_A_)/F), where F_A_ represents the molar flow of C_3_H_8_ at Δ*t*. Repeated reactions with different samples from the same batch of catalyst delivered raw data that were reproducible to within ±7 %.
