# Supplementary material for: Novel and Effective Copper–Aluminum Propane Dehydrogenation Catalysts
Source: Chemistry. 2011 Sep 21;17(44):12254–6. doi: 10.1002/chem.201102580 (PMC3504978; doi:10.1002/chem.201102580)
Supplement: Supplementary file 1 [file chem0017-12254-sd1.pdf]

CHEMISTRY
A EUROPEAN JOURNAL

Supporting Information

© Copyright Wiley-VCH Verlag GmbH & Co. KGaA, 69451 Weinheim, 2011

Novel and Effective Copper–Aluminum Propane Dehydrogenation Catalysts

Jana Schäferhans,[b] Santiago Gómez-Quero,[a] Daria V. Andreeva,*[b] and Gadi Rothenberg*[a]

chem_201102580_sm_miscellaneous_information.pdf

Experimental Section

Materials and Instrumentation

Transmission and scanning electron microscopy (TEM, Zeiss 922 EFTEM operating at 200 kV and LEO 1530 FE-SEM, Zeiss, respectively), in combination with an ultra microtome (Ultracut E Reichert Jung, thickness 50 nm) were applied to characterize the optical response, structure, and size of the Al-Cu powder.

Powder X-ray diffraction (PXRD) diagrams were acquired using a Stoe STADI P X-ray Transmission diffractometer: Cu Ka_1, irradiation at ambient temperature, with 2? = 5–90º.

Surface area and pore size distribution based on physisorption (adsorption and desorption of gases) were measured by the BET[1] and BJH[2] methods using N_2 at 77 K on the vacuum gas sorption Surfer (Thermo Scientific). All samples were dried under vacuum for 24 h at 300°C prior to each measurement.

Al shots, irregular, 15 mm and down, 99.9% (metals basis) from Alfa Aesar and Cu beads, 2-8 mm, 99.9995% (trace metals basis) from Sigma Aldrich were used as received. The water was purified before use in a three stage Millipore Milli-Q Plus 185 purification system and had a resistivity higher than 18.2 M? m·cm. All gases were supplied by Praxair (=99.995% pure). The H_2, Ar and O_2 streams were further purified over molecular sieves and/or BTS columns at 80°C prior to use.

Procedure for synthesising the Pt-Sn/Al_2O_3 reference catalyst

The bimetallic Pt-Sn/Al_2O_3 was prepared by simultaneous addition of the precursor solutions (1 mol% loading of each metal). First, 13.0 cm^3 of an acidified 15 mM $SnCl_2$•$2H_2O$ solution was added to 59.0 cm^3 of a 3 mM H_2PtCl_6•$6H_2O$ solution. Then, 2.0 g of support were added and the volume was adjusted to 200 cm^3. The resulting slurry was heated to dryness at 80°C (2°C min^{-1}) under an Ar purge. The solid residue was dried overnight in an Ar stream (60 cm^3 min^{-1}) at 80°C (heating ramp of 2°C min^{-1}), then cooled to room temperature and stored in a desiccator in the dark. Prior to catalysis, all catalyst samples were ground and sieved to <200 µm and activated in 80 cm^3 min^{-1} 25% v/v H_2/Ar to 600°C (5°C min^{-1}) for 1 h.

References

[1] S. Brunauer, L. S. Deming, W. E. Deming, E. Teller, *J. Am. Chem. Soc.* **1940**, *62*, 1723-1732.

[2] E. P. Barrett, L. G. Joyner, P. P. Halenda, *J. Am. Chem. Soc.* **1951**, *73*, 373-380.
